# Consciring subjects: Q methodology described

**DOI:** 10.4102/hsag.v25i0.1163

**Published:** 2020-04-02

**Authors:** Ntsandeni Moseya, Solomon Mashegoane, Saraswathie Govender, Malose Makhubela

**Affiliations:** 1 Department of Psychology, Faculty of Humanities, University of Limpopo, Polokwane, South Africa; 2 Department of Psychology, Faculty of Humanities, University of Johannesburg, Johannesburg, South Africa

**Keywords:** concourse, epilepsy, factor array, P-set, Q-sample, Q-sorting

## Abstract

**Background:**

Despite the availability of Q methodology as a qualitative research alternative that seemingly circumvents the limits of standard qualitative methods across various fields, a recent review of qualitative research literature in leading health-related South African journals indicated that Q methodology is hardly a method of choice in South Africa.

**Aim:**

This article demonstrates the application of Q methodology, a qualitative research option, in psychological research. The methodology is suitably designed to investigate and clarify diverse subjective experiences, attitudes, opinions and/or beliefs held by a group of people on a given topic.

**Methodology:**

A study on the subjective understandings and perceptions of epilepsy is used to illustrate how Q methodology works. In this particular study, a diverse group of participants, comprising students, traditional healers, doctors, nurses, pastors, high school teachers, laypeople domiciled in rural and urban areas, and speakers of two of the dominant African dialects in the area, was used.

**Results:**

Analysis produced three distinctive factors that are appositely named the scientific, the moderated traditionalist and the community-oriented stances. Each factor, constituted on the basis of close resemblance and statistical association between the rank orderings, represents an identifiable understanding of epilepsy by an exclusive grouping of participants.

**Conclusion:**

Concluding remarks about Q methodology are provided.

## Introduction

A 10-year (2007–2017) period inspection of entries in leading health-related South African journals, namely, *Curationis, Health SA Gesondheid, South African Journal of Psychology* and the *South African Medical Journal*, did not find a single study using Q methodology as a method of choice. *Health SA Gesondheid* published 313 articles in the said period, of which 43% were qualitative. Yet, none of the latter featured Q methodology. A study by Oberholzer et al. (2011:4) used a modified form of Q sorting. However, it was not considered an exemplary Q methodology study as it did not incorporate all the elements of Q methodology (see Stainton Rogers 1995:185–186). Our contribution hopes to develop interest in Q methodology by illustrating its use, in the process describing its mechanics of subjectivity.

Q methodology is a subjective procedure used to explore existing perspectives on a topic that can potentially elicit a range of distinct points of view. Unlike other standard qualitative procedures, it blends qualitative and quantitative techniques to portray operant subjectivity (Brown 1980, 1993; McKeown & Thomas 2013; Watts & Stenner 2012). The procedure was introduced by Stephenson in the 1930s as an organised context for individuals to provide their own perspectives on a given topic, allowing the points of view to be objectively analysed and compared once they are provided (Brown 1980:8–9; Stephenson 1953:4). Indeed, Q methodology takes advantage of man’s propensity to reorder and configure according to one’s perception what initially seems to be unordered, discrete stimuli (also see Watts & Stenner 2005:76).

The product of reordering stimuli is, according to Brown (1980:18), operant because it exists naturally and takes its meaning from the specific context where it was composed. In the case of Q methodology, it will be shown later that a Q sort is operant in the sense that the individual constructing it is directed by a set of statements available for sorting; one statement is preferred over another, and there is no right or wrong sorting as the individual sorts according to their point of view (Brown 1980:6). Subsequent to sorting, Q methodology goes on to exploit the communicability of the inherent subjectivity. A respondent sorts statements according to some ‘condition of instruction’, what Coogan and Herrington (2011:25) called the sorting ‘terms of reference’. Once the sorting is completed, the Q sort constitutes a characteristic configuration amenable to statistical analysis, making the individual sorter a variable rather than the Q sort itself. Q methodology then has the capacity to hold ‘constant for inspection and comparison’ (Coogan & Herrington 2011:24) the individual’s representation of their viewpoint on an issue.

There are essentially two aspects to Q methodology: the qualitative element is in plain view as the individual configures and forms a standpoint, and the quantitative element is in the background, out of sight, yet offering substantive support to the qualitative element. The entire process takes place in a series of logical steps. In this contribution, the steps followed in conducting a Q methodology study will be described. A study of different viewpoints, attitudes and beliefs about epilepsy will be used as an example.

## Epilepsy as a candidate for study through Q methodology

Epilepsy is a common syndrome of different cerebral disorders of the central nervous system. It is characterised by chronic recurrent but unpredictable seizures arising from an underlying brain abnormality, in particular spurts of electrical discharge in either specific or general areas of the brain. It affects approximately 69 million people worldwide, 10–15 million of whom are from Africa (cf. Jäger et al. 2005:603; Ngugi et al. 2010; Paul et al. 2012).

Although modern scientific knowledge is advanced and cheap effective medical interventions are available (Elger & Schmidt 2008:523; Heaney & Sander 2007:465), many individuals with epilepsy and their next of kin in Africa are less inclined to consult professionals and specialists. Aside from using established traditional remedies such as *sehlare sa seebana* (see Jäger et al. 2005), they seek alternative cures and interventions, and in extreme cases patients isolate themselves or are segregated and abandoned by family and community members (Doganavsargil et al. 2017; Jilek-Aall et al. 1997; Keikelame et al. 2017:3–5; Zoli et al. 2003:40).

At the core of the reaction to epilepsy are contrasting beliefs about causation, disease course and treatment. Probable causes and risk factors for epilepsy are diverse, including malnutrition (Crepin et al. 2007:1932; Gomes, Oliveira & Castro 2011:27–28), malaria (Carter et al. 2004:980) and vascular diseases (Diop et al. 2003:151). Many cases in sub-Saharan Africa and other underdeveloped regions are attributable to cysticercosis (Millogo, Njamnshi & Kabwa-PierreLuabeya 2019:32; Zoli et al. 2003:36–37), a situation which is reversed in the event that safe pig husbandry is practised (Nkouawa et al. 2015:118). Nevertheless, there are many instances where the cause of the disease cannot be identified (Gripper & Welburn 2017:220). Studies in Africa have not been able to identify the cause in more than 50% of the cases (Preux & Druet-Cabanac 2005:25). When the causes are so diverse and causal uncertainty predominates in equally more cases, then there is room for speculation in people’s minds.

Apart from individuals who have never heard of the disease, there are those who believe that epilepsy has supernatural causes and is rightly controlled and remedied through traditional indigenous or religious methods (Devinsky 2003:76–77; Mbewe et al. 2007:456; Millogo et al. 2004:252). They stand in contrast to those who hold scientific beliefs about the causes and treatments of the illness. Knowledge, beliefs, attitudes and behaviour vary in society, with demographic variables such as age, education and domicile responsible for the variance (Bigelowa et al. 2015:30; Ibinga et al. 2015:141; Pupillo et al. 2014:43). Because of the contrasts in understanding and the divergent perceptions, epilepsy is a good candidate for Q methodology study and is an appropriate concept to illustrate the workings of the approach.

## Description of Q methodology

### Background of the study

An exploration of the perspectives on epilepsy was conducted in the Polokwane (Limpopo) area among African individuals from different walks of life. Before proceeding with the study, it was approved by the University of Limpopo’s Faculty of Humanities research and ethics committee (details can be found in Moseya 2009). Participation was restricted to black Africans so as to, at the least, control for culture. Selected participants were presumed to potentially hold different views about epilepsy in general. This is informed by the epilepsy literature that suggests that people from different backgrounds (domicile, class, education and so on) hold different attitudes and perceptions towards the illness (Ekeh & Ekrikpo 2015). The first step in designing the study was to conceive of the nature of the problem to be investigated. Unlike in typical hypothetico-deductive studies common in quantitative research, a Q methodology study formulates a research question. The research question, a straightforward, single-proposition statement, was used to guide the formulation and structuring of the Q set, and eventually formed the basis for the ‘condition of instruction’. The study simply explored the question of existing opinions on epilepsy. As the intention was to uncover the existing opinions about the condition, the study ensured that participants were diverse, as will be explained immediately hereafter.

### P-set

Participants who are carefully selected for their theoretical relevance to the problem under consideration are referred to as a P-set. In quantitative designs sample size can be prescribed for one reason or another, while it is not the case in Q methodology. A P-set of 40–60 is found adequate for all purposes (Brown 1980:260, 1993:104; Watts & Stenner 2012:70–73). However, the determining factor regarding the size of the P-set is whether all existing viewpoints have been adequately covered. Thus, fewer or more people can be used, and single case studies are possible (Brown 1980:58, 1993:94). A formal sampling term closer to the activity of selecting participants for a Q methodological study is ‘maximum variation sampling’ – a purposive sampling method used to capture a wide range of perspectives relating to a subject matter, and to purposefully ensure diversity or heterogeneity in the sample or cases. In this study, 33 participants were identified for inclusion in the P-set. Groups of three participants represented categories of people who in our view may have had some dealings with an epileptic case or hold an opinion about the disease. There were 11 categories, for a total of 33 participants. The categories included profession (teacher, health sciences student, non-health sciences student, doctor, nurse, traditional healer and pastor), domicile, (non-professional urban dweller and non-professional rural dweller) and ethnic group (a traditional Tshivenda speaker and a traditional Sepedi speaker).

### Devising the concourse or Q-population, and the Q-set

A population in a Q methodology study is not a group of respondents as is the case in other traditional methods of research. It is a universe of items (statements, single words, objects, images and so on) relating to the topic of interest, and the sum total is called a concourse or Q-population. It is from the concourse that the final, representative set of items, the Q-set/Q-sample, will be drawn. In the epilepsy study, initial interviews were conducted with diverse individuals to gather statements pertaining to the concept of epilepsy. Of course, a concourse can never be complete. The aim in putting it together is to ensure that at least the most common and diversely representative ideas are covered. The transcribed interviews were carefully scanned to locate any expression of opinion, attitudes and beliefs about epilepsy. The epilepsy literature was also consulted to add any pertinent perspectives that may not have arisen from the interviews. In this way, 137 statements were developed. The statements were hybrids because they were constructed from more than one source, including naturalistic and non-human sources (McKeown & Thomas 2013:21). They were spread across three broad themes, namely, scientific, supernatural (non-religious and religious) and social. Three of the authors finalised and approved 36 statements to form the Q-set. This was performed by analysing and refining the content of the statements to ensure concourse representativeness, clarity and appropriateness to context.

### Q-sorting

**FIGURE 1:**
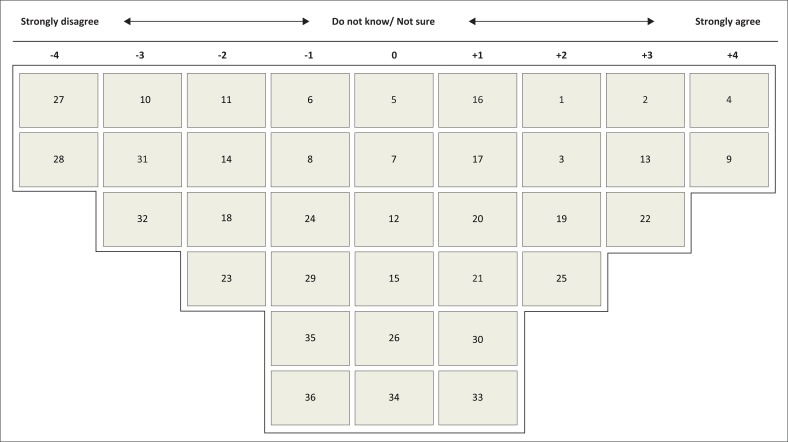
Post-sorting Q sort grid.

Participants were presented with a Q-pack containing the main materials of the study: randomly numbered cards with individually printed statements (Q-set); a quasi-normal distribution grid (i.e. a grid typically used to rank-order statements along a continuum ranging between two extremes; see Figure 1 for a post-sorting example), to record agreement or disagreement with the statements on a nine-point ranking distribution, a demographic information section and a section where participants could write comments (Q-grid); an instruction sheet explaining the sorting process; and a consent form that also included participant rights as research subjects as well as a brief explanation of the nature and aim of the study.

The cards were presented face up, showing the statements to be sorted. The card number was thus hidden from view. Instructions directed participants to carefully compare, sort and then rank-order all 36 statements according to how they agreed or disagreed with them. To be exact, each participant was directed to sort the statements into three piles of ‘agree’, ‘disagree’ and ‘neutral’. Thereafter, they sorted by ranking and ordering the piles into a quasi-normal distribution. After sorting the statements, participants were directed to then record their Q sort using the numbers at the back of the cards. A sorting grid populated with a Q sort array of factor one would look like Figure 1. Post-sorting interviews were conducted to clarify the statement rankings, for instance, why statements were ranked very low or very high, and to allow participants to express any additional view they considered important for the study. In the case of the study used here, no substantive post-interview information emerged.

### Q methodological analysis

The quantitative analysis component in this study involved the use of correlation and factor analysis to group individual participants who express a common view by sorting the statements into nearly the same configuration. Statistical Package for the Social Sciences (SPSS) was used for the purpose. Analysis was carried out for persons rather than by variables (i.e. data were extracted with by-person factor analysis), which means that it was persons’ viewpoints that were correlated rather than the conventional Pearson R standard of correlating tests. Thus, factor analysis in Q methodological analysis goes beyond the mere analysis of a transposed correlation matrix (Brown 1980:19). It is in fact an analysis of a matrix that is centred on the viewpoints of persons in relation to how they rank-order statements or objects.

Q-methodological analysis can be performed on dedicated software. PQMethod 2.35 (Schmolck 2002) is a free program (supplied with a manual) that can be used to conduct all required Q methodology analyses. PCQ (Stricklin & Almeida 2004) is a commercially available Q methodology software. General software such as SPSS can also be used. In the epilepsy study, the statistics and scores that were derived as requirements for Q methodology analysis were the following:

- A correlation analysis was carried out to produce a by-person matrix, consisting of all paired correlations of the 33 Q sorts.
- The correlation matrix was subjected to factor analysis, which in turn produced rotated factor loadings. If correlations show resemblance between two Q sorts, factor loadings are larger, family resemblances of Q sorts. Factor loadings are types of groupings to which people have grouped themselves by virtue of their Q sorts. In our study of the different viewpoints on epilepsy, we introduced the currently *en vogue* and presumably more precise minimum average partial (MAP) test (Velicer 1976) and parallel analysis (PA; Horn 1965) to determine the number of factors and therefore find a statistically precise factor solution. The MAP test and PA both suggested three factors. For instance, a minimum average squared correlation of *r*^2^ = 0.034 was achieved for the three-component solution with Velicer’s MAP (1976) test. Principal components analysis with Varimax rotation was then conducted, and factor extraction was limited to three factors.
- Factor weighting was conducted to reflect the actual contribution of a Q sort to the factor it loads on. Alternatively, a factor loading is an approximation of how much a Q sort resembles the factor it loads to (Brown 1980). Given that there is variance in how close Q sorts resemble their respective factors, weighting was a necessary step in the study of viewpoints on epilepsy before the merging of related Q sorts was executed. Weighting was achieved using Spearman’s (1927, cf. Brown 1980:241–242; McKeown & Thomas 2013:132) formula:
  $$w=\frac{f}{1-f^{2}}$$ [Eqn 1]
  where *w* is the weight and *f* is the factor loading.
- Q sorts that loaded on the same factor were merged into a composite factor array. Because more than one factor was extracted, the process applied to each of the factors. The factor arrays were z-transformed to make comparability possible.
- The final step in the analysis was to transform the scores of each of the factor arrays back to the original values of the quasi-normal distribution. The result was factor arrays that resembled Q sorts. Each factor array is constituted by Q sorts that are highly associated with each other and are largely distinct to the Q sorts that belong to other factor arrays. Watts and Stenner (2005:82) call them ‘best estimate’ Q sorts. The sum of factor arrays is in fact a record of the respondents’ interpretive configuration of the Q sample.

## Factor interpretation

Interpretation was conducted to highlight and explain the viewpoint shared by respondents whose Q sorts are correlated and load on the same factor. That shared perspective is embodied in each of the three composite Q sorts or arrays configured in this study (Table 1). The approach was to identify characterising statements of the factors. These are statements that have been ranked high on either end of the distribution grid, that is, +4 and −4, followed by +3 and −3. The first factor was formed of individuals whose viewpoints about epilepsy consisted of conventional, medically informed opinions. Thus, it was named the ‘scientific viewpoint’. It does not come as a surprise that nearly all the Q sorts completed by health professionals loaded on this factor. Participants in the ‘scientific viewpoint’ considered it important that people with epilepsy must take their medication and felt that they should be discouraged from using alcoholic beverages (#4, +4; #9, +4). Thus, they endorsed the view that it was important for people with epilepsy to take care of themselves (#13, +3). They rejected the supernatural causal frame (#27, −4; #28, −4), together with any explanation or viewpoint associated with it (#31, −3; #32, −3).

**TABLE 1:** Final factors.

Item Number	Item	F1	F2	F3
1	If anyone in the family had previously suffered from epilepsy, it is likely that another person in that family will also suffer from the disease.	2	−1	3
2	I understand that if someone is an epileptic, she/he must be treated like any other person with any other type of disability.	3	3	1
3	I understand that epilepsy can be caused by an injury to the head.	2	1	1
4	It is important that epileptics must not default or forget to take their medication.	4	2	2
5	The type of food that we eat can sometimes affect us and may cause epilepsy.	0	−1	0
6	Epilepsy only affects people who have many personal problems.	−1	0	2
7	I think that people with epilepsy also have diabetes.	0	−2	−1
8	When I was growing up as a child, I was always told that epilepsy can be contracted from pets and wild animals.	−1	0	−1
9	I would like to encourage epileptics not to drink alcohol.	4	3	3
10	I think that epileptics are people who are mad or mentally disturbed.	−3	−3	−3
11	I believe that epilepsy is contagious.	−2	−2	−4
12	In children, epilepsy can be seen as mental retardation.	0	1	1
13	I think it is important for epileptics to take care of themselves.	3	1	1
14	It is important that epileptics remain indoors and be given 24-h care.	−2	0	0
15	When I first saw the actions and behaviours of an epileptic, I was scared, and thought of many things such as the person having committed an abortion.	0	−4	−2
16	It is a problem for me to help an epileptic because I am not trained on what to do if such a person falls down.	1	1	−2
17	Most people tend to avoid epileptics because they are afraid that the epileptics will die in their hands.	1	0	1
18	I do not think that I can allow my child to marry an epileptic.	−2	−2	0
19	Epilepsy is very traumatic to both the patients and the people who take care of them.	2	1	4
20	Epileptics are usually not understood by the community.	1	2	4
21	Epileptics tend to have a problem of not socialising with other people.	1	−1	2
22	I think programmes must be organised to teach our community about epilepsy.	3	3	3
23	I think that people who fall down and collapse are faking epilepsy, seeking to draw attention to themselves.	−2	−4	−3
24	Sometimes epilepsy can occur as a result of breaking taboos and societal norms.	−1	0	−2
25	I do not think epilepsy is a result of witchcraft.	2	−1	−1
26	Most people believe that epilepsy can be treated by traditional healers.	0	4	0
27	Epileptics are persons who have been overpowered by a *tokološe*.	−4	−3	0
28	I think epileptic actions are caused by a powerful devil dwelling in the river.	−4	−2	1
29	I believe that epilepsy is curable with African treatment methods.	−1	2	−2
30	According to African culture, epileptics are viewed as having spirit visitations.	1	−1	−1
31	I believe that if an epileptic has accidentally fallen into a fire or water before, the illness cannot be cured.	−3	4	−3
32	I believe that epileptics have worms in their heads.	−3	−1	−4
33	When we pray for epileptics, we are not performing miracles, but we call upon God’s power to heal the illness.	1	2	2
34	Sometimes it happens that a person who has never suffered from epilepsy may develop epileptic attacks during prayer.	0	−3	−1
35	I think that epileptics have contracted this illness by staying in or passing through a place or a road haunted by spirits.	−1	0	−1
36	Epileptics must drink blessed water, or bathe or recite from holy texts for healing.	−1	1	0

F, factor.

The second factor was named a ‘moderated traditionalist viewpoint’ because the participants endorsed traditional healing (#26, +4; #29, +3) and the indirect involvement of supernatural forces in the cure of epilepsy (#31, +4), and appeared to reject genetic factors in the disease (#1, −1). Yet, they are open to education about epilepsy (#22, +3) and are negative about any type of supernatural causal explanations (#27, −3; #34, −3). Their position has to do with the cures rather than the causes of the disease. Regardless of their traditionalist viewpoint, they would discourage alcohol intake among people with epilepsy (#9, +3). Although the taking of medication was not a distinguishing characteristic in this factor, it nevertheless was endorsed (#4, +2). Participants in the ‘moderated traditionalist viewpoint’ do not seem to react with typical bigotry such as being shocked and overreacting to witnessing an epileptic attack (#15, −4; #23, −4). Interestingly, traditional healers belong to this factor.

The third and final factor is called the ‘community-oriented viewpoint’ because its focus is on the socio-cultural aspects of epilepsy. Participants in this factor are of the view that epilepsy is traumatic to patients and their caregivers (#19, +4) and they are concerned that people with epilepsy are not well understood by community members (#20, +4). They strongly disagree with the ideas that epilepsy is a contagious mental disturbance (#10, −3; #11, −4), that those afflicted can seek attention by faking seizure attacks (#23, −3) and that its cure is linked to supernatural events. All the Q sorts completed by pastors (all of whom were affiliated to charismatic churches) belong to this array. However, the array also had a nurse, a health sciences student and a teacher.

There were only two clear consensus statements in this study. Participants in all factors agreed with the statement pertaining to the institution of programmes to educate the community about epilepsy (#22, +3) and disagreed with the description that people with epilepsy are mad (#10, −3). Nonetheless, a distinguishing feature about the group of participants who took part in this study is that they tended to share a number of characteristics. For instance, factors 2 and 3 participants agreed with the explanation that epilepsy can be caused by head trauma, a distinguishing feature of factor 1. It is clear from the results of the present study that the context in which this study was conducted is one where the modern, scientific view of epilepsy is making inroads. It is likely that awareness programmes and other forms of intervention have eroded the traditionalist view of epilepsy, where the causes and interventions of the disease are linked exclusively to supernatural causes.

Another interesting observation about this study is the workings of the respondents’ operant subjectivity. Doubt about the participants’ freedom to impose their understanding is dispelled, and the disappearance of the researcher’s intended meaning or understanding of the statements is evident. A statement (Item 32: ‘I believe that epileptics have worms in their heads’.), which was formed on the basis of the role of neurocysticercosis and other parasitic infections of the brain in epilepsy (Reddy & Volkmer II 2017:177), has been rated negatively by all respondents including those with a scientific orientation to epilepsy. We suspect that they assumed a literal, lay interpretation of the scientific notion conveyed. They appeared oblivious to the fact that neurocysticercosis, an infectious cause of epilepsy, is actually itself caused by brain cysts from the *Taenia solium* tapeworms (Millogo et al. 2019:32; Reddy & Volkmer II 2017:177; Zoli et al. 2003:37).

## Evaluation of Q methodology

There are weaknesses related to Q methodology. McKeown (1990:3) did observe that studies of subjectivity have to contend with the play of two subjectivities, that of the observer and the observed. Q methodology attempts to tame the subjectivity of the observer and prioritise that of the observed. Nonetheless, there is always the danger of the observer’s subjectivity creeping into the process. The latitude given to the researcher to decide on the final concourse allows for purposeful structuring. However, it can allow the researcher’s subjectivity and bias to creep in. The same problem is possible during analysis and interpretation. In spite of Q methodologists following rules of analysis and introducing standards of interpretation, the exercises remain an art. They permit the researcher to make a judgement about the type of factor analysis to use, and depending on the analytic procedure, the number of factors to extract. Researchers can even decide on additional items to interpret over and above the factor characterising items. Awareness of the possible role of the researcher’s subjectivity will, as is the case in all research paradigms, go a long way in minimising its intrusion at any stage of the research process.

Another limitation has to do with how far the results can be generalised beyond the context in which they were made. Q methodology findings have the same generalisability limitations as conventional qualitative research methods. Research data produced from Q methodology are limited because of its somewhat exploratory nature and its data not being based on random samples. Nevertheless, the latter is not a goal of the method (Brown 1980:192). It only illuminates the viewpoints that are there in a given context and time without establishing the actual proportion of people holding them.

On the positive side, Q methodology is unique in that it is able to characterise the gestalt of each individual participant’s response, and then integrate it into a configuration common to other respondents sharing the viewpoint. Thus, it is a suitable and powerful methodology for exploring and explaining patterns in subjectivities, generating new ideas and hypotheses, and identifying contrasts in views, opinions and preferences. Q methodology offers an innovative approach to qualitative analysis through the ‘quantification of patterned subjectivities’ (Shemmings 2006:147). Other qualitative methods can only group participants into themes, but they do not offer the sophisticated configuration that Q methodology allows. Related to this is the meaning that participants attach to the statements, apart from the meanings generated during the stage of constructing the Q sample. Q methodology incorporates the participant’s meaning-making and accepts it as a spontaneous but legitimate act in the research process. No wonder many participants enjoy the sorting process and, when completed, express satisfaction with it.

## Conclusion

Q methodology is available as an approach that seemingly circumvents the limits of standard qualitative methods. In particular, the important element of the subjectivity of the researcher is present. Yet, unlike in other methods, Q methodology acknowledges its presence, and the techniques of the methodology are comparatively less amenable to it. The strength of the method lies in its combination of both qualitative and quantitative techniques to harness subjectivity.

## References

[CIT0001] Bigelowa J., Berrett S., Kimuli I. & Katabira E., 2015, ‘Perceptions of epilepsy among first-year medical students at Mulago Hospital in Kampala, Uganda’, *Epilepsy & Behavior* 51, 28–32. 10.1016/j.yebeh.2015.06.020 26253598

[CIT0002] Brown S., 1993, ‘A primer on Q methodology’, *Operant Subjectivity* 16(3/4), 90–138.

[CIT0003] Brown S.R., 1980, *Political subjectivity: Applications of Q methodology in political science*, Yale University Press, New Haven, CT.

[CIT0004] Carter J.A., Neville B.G.R., White S., Ross A.J., Otieono G., Mturi N. et al., 2004, ‘Increased prevalence of epilepsy associated with severe falciparum malaria in children’, *Epilepsia* 45(8), 978–981. 10.1111/j.0013-9580.2004.65103.x 15270766

[CIT0005] Coogan J. & Herrington N., 2011, ‘Q methodology: An overview’, *Research in Secondary Teacher Education* 1(2), 24–28.

[CIT0006] Crepin S., Houinato D., Nawana B., Avode G.D., Preux P.-M. & Desport J.-C., 2007, ‘Link between epilepsy and malnutrition in a rural area of Benin’, *Epilepsia* 48(10), 1926–1933. 10.1111/j.1528-1167.2007.01159.x 17565592

[CIT0007] Devinsky O., 2003, ‘Religious experiences and epilepsy’, *Epilepsy & Behavior* 4(1), 76–77. 10.1016/S1525-5050(02)00680-7 12609231

[CIT0008] Diop A.G., de Boer H.M., Mandhlate C., Prilipko L. & Meinardi H., 2003, ‘The global campaign against epilepsy in Africa’, *Acta Tropica* 87(1), 149–159. 10.1016/S0001-706X(03)00038-X 12781390

[CIT0009] Doganavsargil O, Cinemre B., Senol Y., Barcin E. & Gokmen Z., 2017, ‘Epilepsy and stigmatization in Turkey’, *Epilepsy & Behaviour* 73, 100–105. 10.1016/j.yebeh.2017.05.015 28623751

[CIT0010] Ekeh B.C. & Ekrikpo U.E., 2015, ‘The knowledge, attitude, and perception towards epilepsy amongst medical students in Uyo, Southern Nigeria’, *Advances in Medicine* 2015(876135), 6 10.1155/2015/876135 PMC4590962 26556558

[CIT0011] Elger C.E. & Schmidt D., 2008, ‘Modern management of epilepsy: A practical approach’, *Epilepsy & Behavior* 12(4), 501–539. 10.1016/j.yebeh.2008.01.003 18314396

[CIT0012] Gomes T.K.C., Oliveira S.L. & Castro R.M., 2011, ‘Malnutrition and experimental epilepsy’, *Journal of Epilepsy & Clinical Neurophysiology* 17(1), 24–29. 10.1590/S1676-26492011000100006

[CIT0013] Gripper B.G. & Welburn S.C., 2017, ‘Neurocysticercosis infection and disease: A review’, *Acta Tropica* 166, 218–224. 10.1016/j.actatropica.2016.11.015 27880878

[CIT0014] Heaney D.C. & Sander J.W., 2007, ‘Antiepileptic drugs: Generic versus branded treatments’, *Lancet Neurology* 6(5), 465–468. 10.1016/S1474-4422(07)70105-9 17434101

[CIT0015] Horn J.L., 1965, ‘A rationale and test for the number of factors in factor analysis’, *Psychometrika* 30(2), 179–185. 10.1007/BF02289447 14306381

[CIT0016] Ibinga E., Ngoungou E.B., Olliac B., Hounsossou C.H., Dalmay F., Mouangue G. et al., 2015, ‘Impact of epilepsy on children and parents in Gabon’, *Epilepsy & Behavior* 44, 110–116. 10.1016/j.yebeh.2014.12.035 25678031

[CIT0017] Jäger A.K., Mohoto S.P., Van Heerden F.R. & Viljoen A.M., 2005, ‘Activity of a traditional South African epilepsy remedy in the GABA-benzodiazepine receptor assay’, *Journal of Ethnopharmacology* 96(3), 603–606. 10.1016/j.jep.2004.10.005 15619585

[CIT0018] Jilek-Aall L., Jilek M., Kaaya J., Mkombachepa L. & Hillary K., 1997, ‘Psychosocial study of epilepsy in Africa’, *Social Science & Medicine* 45(5), 783–795. 10.1016/S0277-9536(96)00414-5 9226801

[CIT0019] Keikelame M.J., Suliaman T., Hendriksz M. & Swartz L., 2017, ‘Psychosocial challenges affecting the quality of life in adults with epilepsy and their carers in Africa: A review of published evidence between 1994 and 2014’, *African Journal of Primary Health Care Family Medicine* 9(1), a1275 10.4102/phcfm.v9i1.1275 PMC5387367 28397523

[CIT0020] Mbewe E., Haworth A., Atadzhanov M., Chomba E. & Birbeck G.L., 2007, ‘Epilepsy-related knowledge, attitudes, and practices among Zambian police officers’, *Epilepsy & Behavior* 10(3), 456–462. 10.1016/j.yebeh.2006.12.010 17363333 PMC2749646

[CIT0021] McKeown B., 1990, ‘Q-methodology, communication and the behavioural text’, *EJC/REC* 1(1), viewed 12 January 2018, from www.cois.org/getfile/MCKEOWN_V1N190ACCESS02/02/2000.

[CIT0022] McKeown B. & Thomas D., 2013, *Q methodology*, 2nd edn., Sage, London.

[CIT0023] Millogo A., Njamnshi A.K. & Kabwa-PierreLuabeya M., 2019, ‘Neurocysticercosis and epilepsy in sub-Saharan Africa’, *Brain Research Bulletin* 145, 30–38. 10.1016/j.brainresbull.2018.08.011 30170188

[CIT0024] Millogo A., Ratsimbazafy V., Nubukpo P., Barro S., Zongo I. & Preux P.M., 2004, ‘Epilepsy and traditional medicine in Bobo-Dioulasso (Burkina Faso)’, *Acta Neurologica Scandinavica* 109(4), 250–254. 10.1111/j.1600-0404.2004.00248.x 15016006

[CIT0025] Moseya N., 2009, ‘Perception of epilepsy: A Q-methodology study’, MA dissertation, Dept of Psychology, University of Limpopo, Polokwane.

[CIT0026] Ngugi A.K., Bottomley C., Kleinschmidt I., Sander J.W. & Newton C.R., 2010, ‘Estimation of the burden of active and life-time epilepsy: A meta-analytic approach’, *Epilepsia* 51(5), 883–890. 10.1111/j.1528-1167.2009.02481.x 20067507 PMC3410521

[CIT0027] Nkouawa A., Dschanou A.R., Moyou-Somo R., Sako Y. & Ito A., 2015, ‘Seroprevalence and risk factors of human cysticercosis and taeniasis prevalence in a highly endemic area of epilepsy in Bangoua, West Cameroon’, *Acta Tropica* 165, 116–120. 10.1016/j.actatropica.2015.12.019 26747010

[CIT0028] Oberholzer A.E., Nel E., Myburgh C.P.H. & Poggenpoel M., 2011, ‘Exploring the needs and resources of children in a haematology-oncology unit’, *Health SA Gesondheid* 16(1), Art. #565, 12 pages. 10.4102/hsag.v16i1.565

[CIT0029] Paul A., Adeloye R., George-Carey I., Grant L. & Chan K.Y., 2012, ‘An estimate of the prevalence of active and life-time epilepsy: A meta-analytic approach’, *Journal of Global Health* 2(2), 1–13. 10.7189/jogh.02.020405 PMC3529318 23289080

[CIT0030] Preux P.-M. & Druet-Cabanac M., 2005, ‘Epidemiology and aetiology of epilepsy in sub-Saharan Africa’, *Lancet Neurology* 4(1), 21–31. 10.1016/S1474-4422(04)00963-9 15620854

[CIT0031] Pupillo E., Vitelli E., Messina P. & Beghi E., 2014, ‘Knowledge and attitudes towards epilepsy in Zambia: A questionnaire survey’, *Epilepsy & Behavior* 34, 42–46. 10.1016/j.yebeh.2014.02.025 24681384

[CIT0032] Reddy D.S. & Volkmer R. II, 2017, ‘Review: Neurocysticercosis as an infectious acquired epilepsy worldwide’, *Seizure: European Journal of Epilepsy* 52, 176–181. 10.1016/j.seizure.2017.10.004 29055271

[CIT0033] Schmolck P., 2002, *PQMethod 2.35 with PQROT 2.0 (10-Nov-2014) [Computer software]*, viewed 22 December 2017, from schmolck.userweb.mwn.de/qmethod/downpqwin.htm.

[CIT0034] Shemmings D., 2006, ‘Quantifying qualitative data: An illustrative example of the use of Q methodology in psychosocial research’, *Qualitative Research in Psychology* 3(2), 147–165. 10.1191/1478088706qp060oa

[CIT0035] Stephenson W., 1953, *The study of behavior: Q-technique and its methodology*, University of Chicago Press, Chicago, IL.

[CIT0036] Stainton Rogers R., 1995, ‘Q Methodology’, in Smith J., Harré R. & Van Langenhove L. (eds.), *Rethinking methods in psychology*, 178–192, Sage, London.

[CIT0037] Stricklin M. & Almeida J., 2004, *PCQ for Windows standard/academic edition [Computer software]*, PCQ Software, Portland, OR.

[CIT0038] Velicer W.F., 1976, ‘Determining the number of components from the matrix of partial correlations’, *Psychometrika* 41(3), 321–327. 10.1007/BF02293557

[CIT0039] Watts S. & Stenner P., 2005, ‘Doing Q methodology: Theory, method and interpretation’, *Qualitative Research in Psychology* 2(1), 67–91. 10.1191/1478088705qp022oa

[CIT0040] Watts S. & Stenner P., 2012, *Doing Q methodological research: Theory, method and interpretation*, Sage, London.

[CIT0041] Zoli A., Shey-Njila O., Assana E., Nguekam J.P., Dorny P., Brandt J. et al., 2003, ‘Regional status, epidemiology and impact of *Taenia solium cysticercosis* in Western and Central Africa’, *Acta Tropica* 87(1), 35–42. 10.1016/S0001-706X(03)00053-6 12781376

